# A Retrospective Analysis of a Cohort of Patients Treated With Immune Checkpoint Blockade in Recurrent/Metastatic Head and Neck Cancer

**DOI:** 10.3389/fonc.2022.761428

**Published:** 2022-01-27

**Authors:** Michel Bila, Jeroen Van Dessel, Maximiliaan Smeets, Vincent Vander Poorten, Sandra Nuyts, Jeroen Meulemans, Paul M. Clement

**Affiliations:** ^1^ Oral and Maxillofacial Surgery, University Hospitals Leuven and OMFS-IMPATH Research Group, Department of Imaging & Pathology, Faculty of Medicine, KU Leuven, Leuven, Belgium; ^2^ Department of Oncology, KU Leuven, Leuven, Belgium; ^3^ Department of General Medical Oncology, Leuven Cancer Institute, University Hospitals Leuven, Leuven, Belgium; ^4^ Otorhinolaryngology Head and Neck Surgery, University Hospitals Leuven, Leuven, Belgium; ^5^ Department of Oncology, Section Head and Neck Oncology, KU Leuven, Leuven, Belgium; ^6^ Radiation Oncology, Department of Oncology, Leuven Cancer Institute, University Hospitals Leuven, Leuven, Belgium

**Keywords:** mouth neoplasms, head and neck neoplasms, squamous cell carcinoma of head and neck (HNSCC), immunotherapy, immune checkpoint inhibitors, programmed cell death 1 (PD-1), Programmed death-ligand 1 (PD-L1), cytotoxic t-lymphocyte-associated protein 4 (CTLA-4)

## Abstract

**Objective:**

The treatment approach of recurrent or metastatic head and neck squamous cell carcinoma (R/M HNSCC) has long been similar for all patients. Any difference in treatment strategy was only based on existing comorbidities and on preferences of the patient and the treating oncologist. The recent advance obtained with immune therapy and more specifically immune checkpoint blockade (ICB) has been a true game changer. Today, patients and physicians have a choice to omit chemotherapy. In a small subset of patients, ICB induces a very durable disease control. The subgroup of patients in which ICB without chemotherapy would be the preferential approach is still ill-defined. Yet, this evolution marks a major step towards a more personalized medicine in R/M HNSCC.

**Materials and Methods:**

In this paper, we present a retrospective cohort study of a patient population that was treated with ICB in a single center and we analyze potential factors that are associated with outcome and may help to select patients for treatment with ICB.

**Results:**

137 consecutively treated patients were identified. Male gender and metastatic disease appeared to be associated with improved overall survival (OS). There was no correlation observed with age, number of previous treatment lines or immune target.

**Conclusion:**

Along with PD-L1 status defined by Combined Positive Score (CPS), clinical parameters such as site of recurrence and gender may help to define the optimal treatment strategy in R/M HNSCC.

## Introduction

The treatment with curative intent of squamous cell carcinoma of the head and neck (HNSCC) has historically been based on surgery and radiation therapy. In recurrent or metastatic disease, platinum-based combination chemotherapy has long been the standard approach for treatment with palliative intent. In the absence of a proven survival benefit, the treatment choice was tailored mainly on comorbidity, costs and individual preferences. The first trial that showed an extra survival benefit in recurrent or metastatic squamous cell carcinoma of the head and neck (R/M HNSCC) introduced the addition of cetuximab to platinum-based chemotherapy (1). Although cetuximab, a monoclonal antibody against epidermal growth factor receptor (EGFR), is defined as a “targeted therapy”, its use has not been restricted to tumors with proven expression of the target, because the vast majority of squamous cell carcinomas of the head and neck is characterized by EGFR amplification (2).

As a consequence, this so-called “EXTREME regimen” has emerged as the standard of care in R/M HNSCC for patients who could tolerate this treatment (3). More recently, immune checkpoint blockade (ICB) has also emerged as a valuable treatment option. The first solid evidence was provided by a randomized trial that showed superiority of the PD-1 antibody nivolumab over best investigator’s choice, in a patient population that had been pretreated with cisplatin (4). Interestingly, the trial not only showed an improvement in median overall survival but was also associated with a better quality of life. Furthermore, it is important to highlight that the long-term survival rate in patients treated with nivolumab was substantially higher than in the control group. This observation is very important, because a substantial improvement in the chance to be alive after two years is clearly more relevant than an average two-month gain in survival probability. Subsequently, another PD-1 antibody, pembrolizumab, has shown similar survival statistics in platinum-pretreated R/M HNSCC, albeit without a statistically significant difference in overall survival compared to chemotherapy. The latter observation is probably due to a substantial subgroup that crossed over to immune therapy from the control group (5). Comparable findings have been reported with the PD-L1 antibody durvalumab, again without a proven benefit over standard of care (6). The addition of the CTLA4-antibody tremelimumab showed no extra added value in that trial.

Pembrolizumab was tested in first line R/M HNSCC, either as single agent or in combination with platinum/5FU, and compared to the standard of care, the EXTREME regimen (7). This trial showed a clearly superior outcome when pembrolizumab was combined with platinum/5FU compared to cetuximab combined with this chemotherapy doublet. Yet, this trial also showed that the chance of benefit was related with the PD-L1 status, calculated as a Combined Positive Score (CPS). Indeed, patients treated in both pembrolizumab-containing arms had a better survival compared to control, if CPS was greater than 1. Although an exact algorithm cannot be defined yet, the possibility to omit chemotherapy has risen, and with that, a giant step towards a more personalized approach in the treatment of this disease.

Next to PD-L1 status, other factors have been proposed that could influence the success of immunotherapy and may help tailoring our choice of treatment. A single-arm phase II study with durvalumab suggests a better prognosis in human papillomavirus- (HPV-) positive squamous cell carcinoma in a PD-1 positive cohort (8). It is hypothesized that HPV may induce a better antigen-presentation and thus enhance the ability of the immune system to target the tumor. Similarly, the cytotoxic effect of chemo- or radiotherapy may also induce more tumor epitopes. This idea has revived the concept of abscopal effects of radiation therapy. The combined use of radiotherapy and checkpoint inhibition is currently being explored in several trials (9).

In this manuscript we provide data that may help to select the best approach for patients presenting with R/M HNSCC. To this purpose we have retrospectively analyzed our own patient cohort with R/M HNSCC for significant factors that may influence treatment decisions or guide future research.

## Materials and Methods

### Study Design and Patient Selection

A retrospective cohort study design was completed following the Strengthening the Reporting of Observational Studies in Epidemiology (STROBE) guidelines (10). The study was approved by the Medical Ethics Committee of the University Hospitals Leuven, Leuven, Belgium. The study sample was derived from a population who presented for immunotherapy of R/M HNSCC at the Department of General Medical Oncology of the University Hospitals Leuven from December 2013 to February 2020. Eligible patients were 18 years of age or older with histologically confirmed diagnosis of R/M HNSCC, not amenable to curative therapy. Patients received a single agent and fixed dose of either PD1, PDL-1 with or without CTLA-4 inhibitors. The number of prior treatments was not a limiting factor for inclusion. Performance status was not a formal selection criterion for ICB treatment in our standard of care practice. Patients who received concurrent chemotherapy or biological therapy were excluded.

### Outcome Parameters

The primary endpoint was overall survival, defined as the time from start of immunotherapy to death due to any cause. Secondary endpoints included overall response rate, progression-free survival, duration and time of response. Tumor response [complete (CR), partial (PR), stable disease (SD) or progression of disease (PD)] was evaluated by Computed Tomography or Magnetic Resonance Imaging. These modalities were not standardized at predefined time points in this patient cohort, but patients had imaging at least every 3 months. Progression-free survival was defined as the time from therapy onset to first documented disease progression according to the Response Evaluation Criteria in Solid Tumors 1.1 (RECIST1.1) criteria (11), evident clinical progression or death due to any cause, whichever occurred first. Duration of response was defined as the time from the first RECIST response until disease progression in patients who achieved a partial or complete response. Time to response was defined as the time from start of immunotherapy to CR or PR.

### Statistical Analysis

The statistical analyses are based on a 24-month data cut-off. The distributions of overall survival and progression-free survival were estimated by the Kaplan-Meier method and compared by means of log-rank tests for therapeutic modality (PD-L1, PD1, CTLA-4 + PD-L1), gender (male, female), age (≤ 65, > 65 years), tumor type (metastatic, locoregional recurrence) and line of palliative treatment (first, second or more) for the entire patient group. The distribution of overall survival for subsequent salvage chemotherapy (yes, no) was calculated on the subgroup of patients with tumor progression after ICB treatment. Cox proportional-hazards models were used to univariately estimate HR and calculate corresponding 2-sided 95% CI’s. Statistical analysis was performed in SPSS (IBM, New York, USA). The significance level α was 0.05 for all tests.

## Results

### Patient Characteristics

The data cut-off date was 13 July 2020, 6 months after the last patient began treatment. The study sample included 137 patients in the final analysis. Twenty-six patients (19%) were still on treatment or were in follow-up at the time of analysis. Two patients (1%) were lost to follow-up. 76% of patients received a PD-1 inhibitor, 15% received a PD-L1 inhibitor and 9% received a PD-L1 inhibitor combined with CTLA-4 inhibitor. The median age was 64 years (range 31 – 84 years), and 78% of patients were male. The majority of patients were either current (56%) or former smokers (41%). Oral cavity was the primary location in most patients (45%), followed by pharynx (34%), larynx (16%) and other anatomical sites in the head and neck region (5%). Patterns of recurrence comprised exclusive locoregional recurrence in 66 patients (48%) and distant metastases with or without locoregional recurrence in 71 patients (52%). Ninety patients (66%) received two or more consecutive lines of systemic therapy for R/M HNSCC and thirty-nine patients (33%) received subsequent salvage chemotherapy after tumor progression under ICB treatment.

### Efficacy

**Figure 1:**
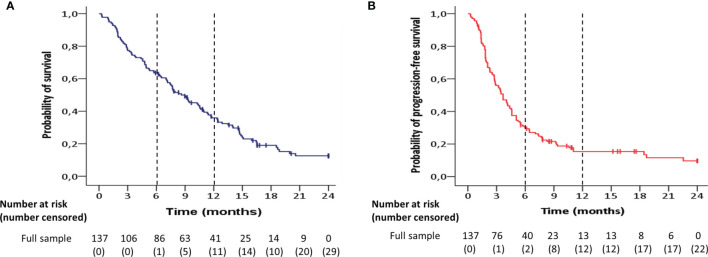
Kaplan-Meier estimates of **(A)** overall survival and **(B)** progression-free survival for the entire patient group (n=137).

The Kaplan-Meier estimate of median overall survival was 9.0 months [95% Confidence Interval (CI) 6.9-11.0] in the entire patient group (**Figure 1A**). Patient survival at 6, 12 and 24 months was 64%, 39% and 16%, respectively. The median progression-free survival was 3.7 months (95% CI 2.9-4.4) for the whole patient group (**Figure 1B**). The proportion of patients with progression-free survival was 31% at 6 months, 10% at 12 months and 4% at 24 months. Median follow-up duration was 28 months (interquartile range (IQR) 17-37 months).

**Figure 2:**
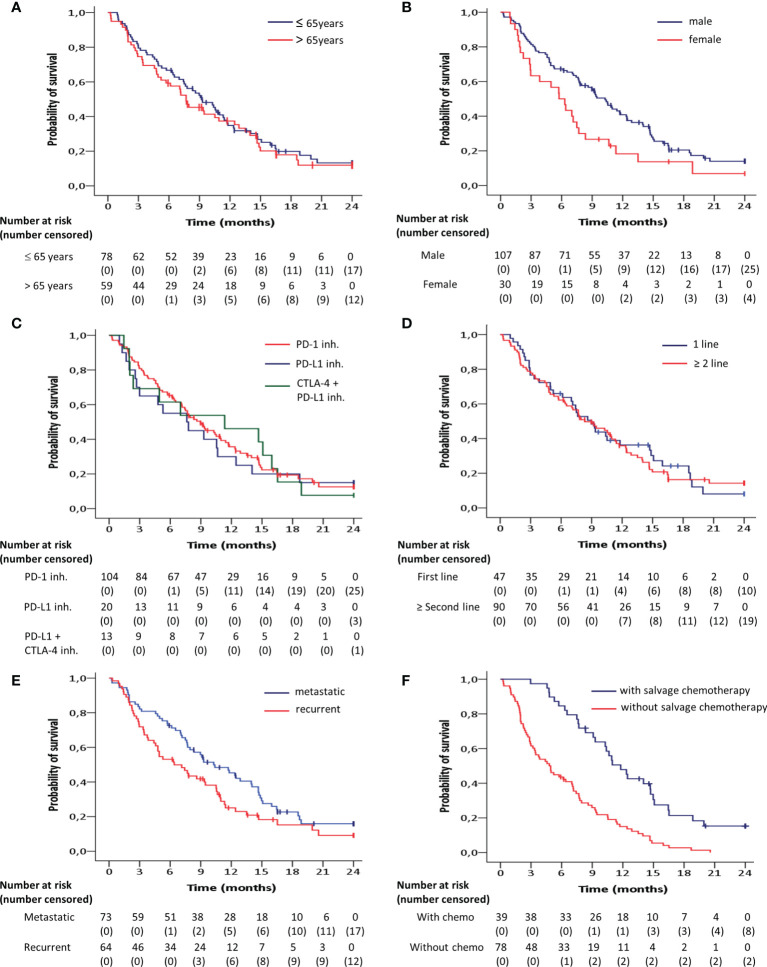
Kaplan-Meier curves for overall survival according to **(A)** age, **(B)** gender, **(C)** target inhibitor, **(D)** line of treatment and **(E)** recurrence pattern, estimated for the entire patient group (n=137). **(F)** Overall survival for the subgroup of patients with tumor progression after immune checkpoint blockade treatment (n=117) depending on whether subsequent salvage chemotherapy was administered. Overall survival was significantly (p<0.05) longer in men compared to women, in metastatic tumors compared to locoregionally recurrent HNSCC and in patients who received subsequent salvage therapy compared to those without.

The median progression-free and overall survival for predefined demographic and clinical subgroups is shown in **Table 1**. Overall survival was significantly longer in men compared to women (HR = 0.59; 95% CI 0.38 – 0.91; p = 0.02) (**Figure 2B**), and in metastatic tumors compared to locoregionally recurrent HNSCC (HR = 0.68; 95% CI 0.46 – 0.99; p = 0.05) (**Figure 2E**). Patients who progressed under ICB treatment and subsequently received chemotherapy showed a significant longer overall survival (HR = 0.35; 95 CI 0.23 – 0.53; p = 0.001) compared to patients without administration of subsequent salvage therapy (**Figure 2F**).

**Table 1:** Overall survival (OS) and progression-free survival (PFS) according to predetermined demographic and clinical subgroups under immune checkpoint blockade treatment.

Variable		Patients	Median OS	Median PFS
		*no. (%)*	*months (95% CI)*	*months (95% CI)*
All patients		137 (100%)	9.0 (6.9 - 11.0)	3.7 (2.9 - 4.4)
Age	≤ 65 years old	59 (43%)	9.3 (6.8 - 11.8)	3.2 (2.3 - 4.1)
	> 65 years old	78 (57%)	7.7 (5.3 - 10.0)	4.6 (3.3 - 5.9)
Gender	Male	107 (78%)	10.3 (8.6 - 12.0)	4.0 (3.2 - 4.9)
	Female	30 (22%)	5.8 (3.8 - 7.7)	2.1 (1.3 - 2.9)
Drug	PD-1	104 (76%)	9.0 (6.7 - 11.1)	3.5 (2.5 - 4.4)
	PD-L1	20 (15%)	7.6 (2.2 - 13.1)	3.7 (2.9 - 4.5)
	CTLA-4 + PD-L1	13 (9%)	11.3 (0 - 22.8)	4.0 (1.6 - 4.4)
Consecutive line of treatment	First	47 (34%)	9.0 (6.9 - 11.1)	3.7 (2.5 - 4.9)
	Second or more	90 (66%)	8.3 (5.6 - 11.1)	3.4 (2.2 - 4.7)
Tumor type	Metastatic	73 (53%)	10.3 (7.0 - 13.5)	4.1 (2.8 - 5.4)
	Recurrent	64 (47%)	6.4 (3.8 - 9.0)	3.4 (2.6 - 4.2)
Salvage chemotherapy	No	78 (67%)	4.8 (3.2 – 6.4)	
after progression	Yes	39 (33%)	11.8 (9.6 – 14.0)	

No significant differences were observed with regard to the overall survival between the age groups at the cut-off of 65 years (HR = 0.89; 95% CI, 0.61 - 1.30; p = 0.55) (**Figure 2A**), between the targets PD-1 and PD-L1 + CTLA-4 (HR = 0.99; 95% CI, 0.54 - 1.81; p = 0.99), between the targets PD-1 and PD-L1 (HR = 1.11; 95% CI, 0.65 – 1.89; p = 0.71) (**Figure 2C**), and between first line of palliative treatment versus second line or more (HR =0.96; 95% CI, 0.65 - 1.43; p = 0.85) (**Figure 2D**).

**Figure 3:**
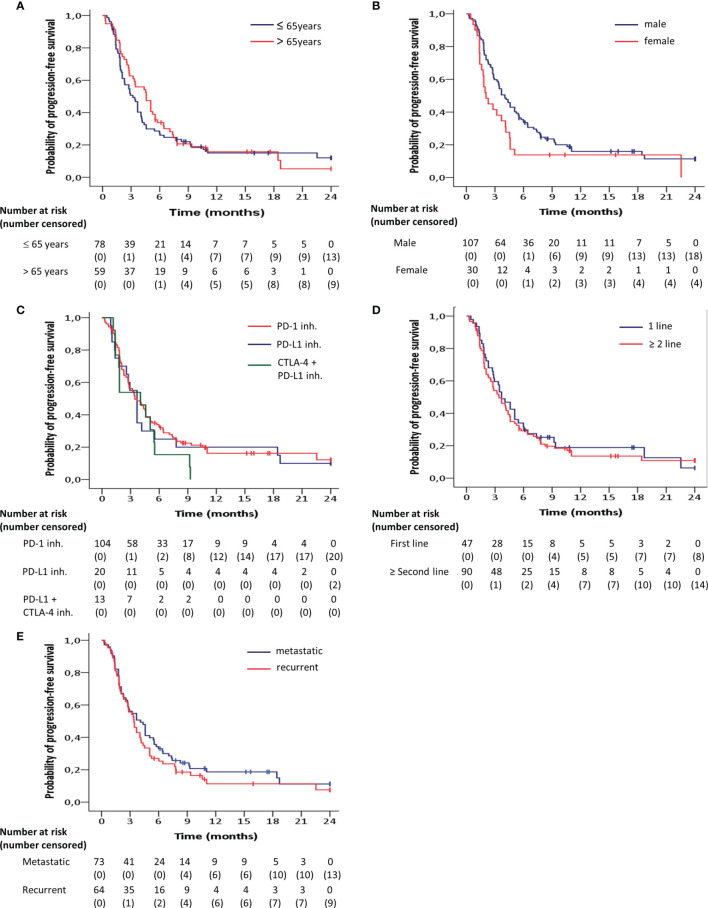
Kaplan-Meier curves for progression-free survival according to **(A)** age, **(B)** gender, **(C)** target inhibitor, **(D)** line of treatment, **(E)** pattern of recurrence, estimated for the entire patient group (n=137). Progression-free survival was significantly (p<0.05) longer in men compared to women.

Progression-free survival was significantly longer in men compared to women (HR = 0.63; 95% CI 0.40 - 0.98; p = 0.04) (**Figure 3B**). There were no significant differences found with regard to the progression-free survival between age groups below or above 65 (HR = 1.13; 95% CI, 0.78 - 1.63; p = 0.49) (**Figure 3A**), between PD-1 and PD-L1 + CTLA-4 inhibitors (HR = 1.47; 95% CI, 0.82 - 2.63; p = 0.20) (**Figure 3C**), between PD-1 and PD-L1 inhibitors (HR = 1.09; 95% CI, 0.65 – 1.82; p = 0.75) (**Figure 3C**), between first line of treatment versus second line or more (HR = 0.90; 95% CI, 0.61 - 1.32; p = 0.59) (**Figure 3D**), or between metastatic or locoregionallly recurrent HNSCC (HR = 0.83; 95% CI, 0.58 - 1.20; p = 0.34) (**Figure 3E**).

The overall response rate in the entire patient cohort was 22% (95% CI, 16-37%). Twenty-eight patients (20%) had achieved a confirmed partial response (PR) and two patients (1%) achieved a complete response (CR). Median time to response was 3.6 months (IQR 2.0 - 5.2), while the median duration of response was 6.4 months (IQR 3.8 – 13.8) for all patients with R/M HNSCC receiving immunotherapy.

## Discussion

Treatment for R/M HNSCC has not changed dramatically over the last decades until the advent of immunotherapy started reshaping our treatment ability very quickly. Tumor profiling of HNSCC for a more targeted treatment approach was essentially absent, with the potential exception of p16 or HPV status in oropharyngeal SCC. Immunotherapy is challenging this “one size fits all” approach. Since the established efficacy of ICB treatment, multiple trials are on the way considering immunotherapy in an earlier stage in the disease process of HNSCC. The early identification of probable responders will be of vital importance for cost-effectiveness of these treatments.

Ferris and colleagues published a subgroup analysis in their trial according PD-1 and HPV status in which preliminary evidence suggested that patients with a tumor PD-L1 expression of 1% or more or p16 positive tumors may have greater effect from ICB, although the survival rate at two years was similar (12). Cohen et al. found – in the same line - that PD-L1 expression on tumor cells and associated immune cells did predict better outcomes for treatment with pembrolizumab (5). The landmark Keynote-048 study was subsequently published in which they reported profound overall survival benefit, particularly in PD-L1 positive tumors, defined by the combined positive score (CPS) (7). This is defined as the number of PD-L1–positive cells (tumor cells, lymphocytes, and macrophages) divided by the total number of tumor cells × 100; a minimum of 100 viable tumor cells must have been present for the specimen to be considered evaluable. Around 85% of HNSCC tumor cells express PD-L1 when measured with CPS (13). CPS has to be viewed as an “enrichment marker”: a CPS score above 20 is associated with a higher chance of benefit from pembrolizumab, but a lower score does not exclude activity. Beyond analytical considerations, PD-1 and PD-L1 expression are known to express spatial and temporal heterogeneity within a tumor as well (14).

Looking at the results in our cohort, a median OS of 9 months is in keeping with the contemporary literature (OS 6.5 – 14.9 months) (7, 8, 12, 15). We did not observe significant differences in OS between three immune checkpoint inhibitors individually, with or without the addition of CTLA-4 inhibition, in this group of R/M HNSCC patients. Different from clinical trials, performance status was not used for inclusion in our clinical practice. Practically, the comparable survival curve in our unselected patient cohort suggests that the benefits observed in the pivotal trials can be extrapolated to patients seen in daily practice.

The significantly better OS in metastatic compared to recurrent HNSCC cases was unanticipated. Interestingly, also in the Keynote-048 trial, the OS benefit of pembrolizumab monotherapy over chemotherapy plus cetuximab seemed to be restricted to metastatic patients (7). This observation suggests a possible association based on biological differences.

A potential explanation lies in the “Tumor mutational burden” (TMB). TMB is a biomarker-based concept and a possible causal factor contributing to the prolonged OS in metastatic HNSCC as previous reports suggest that TMB in metastatic tissues is notably higher than in primary tissues (16). Lin and colleagues observed a correlation between PD-L1 expression and metastatic risk (17). Cold tumors are known to display lower levels of T-cell inflamed signature compared with healthy tissues in the same individual, from which it has been suggested that T-cell exhaustion is a locally active process of carcinogenesis (18). This process and the resulting T-cell exhaustion might hence be different in metastatic locations and it would be useful to assess any difference in tumor inflamed signature (TIS) in a local and metastatic recurrent tumor site. In colorectal cancer for instance, angiogenesis and inflammatory response are shown to be enriched in matched liver metastasis compared to the colorectal primary tumor (19). Furthermore, prior radiotherapy to the site of recurrence might influence the ICB response. Contrary to metastatic lesions, locoregional recurrence typically occurs in a previously irradiated area.

Further detailed subgroup analysis revealed a significantly better OS for male patients. This finding is in line with a meta-analysis reported by Conforti et al. (20) and with the subgroup analysis of the Keynote-048 (7). On the contrary, the earlier work of Lin et al. identified a higher likelihood of PD-L1 expression in female than in male patients (17). In the multivariate analysis, the PD-L1 status only correlated with worse prognosis in males and smokers, in an era when ICB was not available. Taken together, these data suggest that PD-L1 expression is associated with metastasis and poor prognosis, but that particularly male patients with metastatic PD-L1-positive disease may derive benefit from ICB.

Overall, the PFS of 31% at the six-month time point is comparable with results from the Keynote-048 (25-49%) and CheckMate-141 (19.7%) trials (7, 12). The median duration of response of 6.4 months is somewhat lower compared to the published trials (9.7 months in Checkmate-141 and 8 months in Keynote 055) (4, 21, 22). Interestingly, our data confirm the observation in another published cohort of patients treated with salvage chemotherapy after progression under ICB (23). Although the difference in OS compared to patients who did not receive salvage treatment is at least in part explained by selection bias, the observed median OS of 11,8 months is significantly better than what would be expected. This finding supports the use of ICB early in the treatment of HNSCC. Several trials are already exploring ICB treatment in high risk primary HNSCC.

In Europe, the indication of pembrolizumab in first line R/M HNSCC was registered in august 2020 in patients with a CPS score of 1 or higher. This new treatment option poses questions as clinicians have to make the choice whether or not to add chemotherapy to first line ICB in R/M HNSCC. CPS may guide this decision to some extent. Some suggest to use a score above 20 to select patients for single agent ICB, based on the analysis of Keynote-048. The platinum/5FU doublet adds a lot of toxicity, and the benefit in terms of long-term survival is questionable. The main downside of single agent pembrolizumab may be the possibility of fast progression. Therefore, we suggest to treat clinically fit patients with rapidly progressive disease with combined chemo- and immunotherapy. This way, the higher response rate of chemotherapy can be exploited. In frail patients or patients with a less threatening disease progression, we suggest single agent ICB in patients with CPS greater than 1, as it is much better tolerated and offers a better chance to obtain a durable response and disease control.

We report on a single-center, homogeneous population of patients that is treated with ICB without concurrent chemotherapy, with a mature follow-up. Nevertheless, there are some limitations that need to be taken in account when interpreting our findings. First, patients received different ICB treatments and different lines of previous palliative treatment. In current practice, ICB is commonly used as the treatment of choice. Therefore, these observations are representative for patients treated in daily practice. Second, data on HPV status and PD-L1 expression were not available for the entire patient population. Since the literature remains inconclusive on these matters, testing for p16 in other sites than oropharynx is not part of our standard of care routine. Assessment of PD-L1 expression by CPS has only recently become a standard procedure, with the registration of pembrolizumab as part of the first line treatment in R/M HNSCC.

In conclusion, ICB has profoundly changed our ability to treat R/M HNSCC and offers a small subgroup of patients a durable disease control without the need of chemotherapy. Selection of patients for the optimal treatment approach is challenging. Male patients and patients with metastatic disease appear to benefit more from ICB. Along with CPS, and comorbidity, site of recurrence may guide our treatment approach.

## Data Availability Statement

The data that support the findings of this study are available from the corresponding author upon reasonable request.

## Ethics Statement

The studies involving human participants were reviewed and approved by Ethics Committee of the University Hospitals Leuven, Leuven, Belgium. Written informed consent for participation was not required for this study in accordance with the national legislation and the institutional requirements.

## Author Contributions

PC and MB contributed to conception and design of the study. MB and JVD acquired data. MB, JD, and MS performed data analysis. MB, JVD, and PC wrote the first draft of the manuscript. MS wrote sections of the manuscript. VVP, SN, and JM revised the work critically. All authors contributed to manuscript revision, read, and approved the submitted version.

## Conflict of Interest

The authors declare that the research was conducted in the absence of any commercial or financial relationships that could be construed as a potential conflict of interest.

## Publisher’s Note

All claims expressed in this article are solely those of the authors and do not necessarily represent those of their affiliated organizations, or those of the publisher, the editors and the reviewers. Any product that may be evaluated in this article, or claim that may be made by its manufacturer, is not guaranteed or endorsed by the publisher.
